# Supplementary material for: Evolutionarily Conserved Role of Thioredoxin Systems in Determining Longevity
Source: Antioxidants (Basel). 2023 Apr 17;12(4):944. doi: 10.3390/antiox12040944 (PMC10135697; doi:10.3390/antiox12040944)
Supplement: Supplementary file 1 [file antioxidants-12-00944-s001.zip › antioxidants-2313884-supplementary.pdf]

Table S1. Overview of thioredoxin systems in different organisms

Organism	Gene	Gene ID	Location
**Saccharomyces cerevisiae (Yeast)**	*TRX1*	850732	Cytoplasm
	TRX3	850444	Mitochondria
	TRR1	851955	Cytoplasm
	TRR2	856506	Mitochondria
Caenorhabditis elegans **(Worms)**	*trx-1*	181863	Cytoplasm, Nucleus
	trx-2	179434	Mitochondria
	trx-3	187389	Cytoplasm, Nucleus
	trx-4	189905	Cytoplasm
	trx-5	186914	Not determined
	trxr-1	177466	Cytoplasm
	trxr-2	176259	Mitochondria
Drosophila melanogaster **(Flies)**	*TrxT*	31443	Nucleus
	dhd	31444	Nucleus
	Trx-2	34281	Nucleus
	Trxr-1	31760	Cytoplasm, Mitochondria
	Trxr-2	40475	Mitochondria
Mus musculus **(Mice)**	*Txn1/Trx1*	22166	Cytoplasm, Nucleus
	Txn2/Trx2	56551	Mitochondria
	Txnrd1/TrxR1	50493	Cytoplasm
	Txnrd2/TrxR2	26462	Mitochondria
	Txnrd3/TrxR3	232223	Cytoplasm, Nucleus
Homo sapiens **(Humans)**	*TXN*	7295	Cytoplasm, Nucleus
	TXN2	25828	Mitochondria
	TXNRD1	7296	Cytoplasm
	TXNRD2	10587	Mitochondria
	TXNRD3	114112	Cytoplasm, Nucleus
